# Effects of *Lactobacillus* Alone and in Combination With *Saccharomyces* as Alternatives to Antibiotics on Performance and Immunity in Laying Hens

**DOI:** 10.1002/vms3.71120

**Published:** 2026-08-05

**Authors:** Md. Jahangir Alam, Sonia Tabasum Ahmed, Md. Saiful Islam, Mahfuzul Islam, Md. Mahabbat Ali, Sang‐Suk Lee

**Affiliations:** ^1^ Department of Animal Production and Management Faculty of Animal Science and Veterinary Medicine Sher‐e‐Bangla Agricultural University Dhaka Bangladesh; ^2^ Department of Poultry Science Faculty of Animal Science and Veterinary Medicine Sher‐e‐Bangla Agricultural University Dhaka Bangladesh; ^3^ Department of Microbiology & Parasitology Faculty of Animal Science and Veterinary Medicine Sher‐e‐Bangla Agricultural University Dhaka Bangladesh; ^4^ Department of Animal Science & Technology Sunchon National University Suncheon South Korea

**Keywords:** egg production, immunity, *Lactobacillus*, layer chicken, *Saccharomyces cerevisiae*

## Abstract

**Background:**

The removal of antibiotics from poultry feed has created substantial challenges in commercial layer farming, underscoring the need for safe and sustainable alternatives to safeguard flock health and ensure consumer egg safety.

**Objectives:**

This study investigated the effects of *Lactobacillus plantarum* alone and in combination with *Saccharomyces cerevisiae* as alternatives to antibiotics on laying performance, egg quality, blood parameters, immune competence and caecal microbial populations in layer chickens.

**Methods:**

Experimental microorganisms were isolated from yoghurt and fermented corn dough, and two probiotic formulations were prepared: Probiotic 1 (*L. plantarum*) and Probiotic 2; *L. plantarum *+ *S. cerevisiae*). A total of 240 Isa Brown pullets were randomly allocated to four treatments, each with five replicates of twelve birds. The treatments were T1 (control), T2 (flavomycin; 14 mg/L water), T3 (Probiotic 1; 1 mL/L water) and T4 (Probiotic 2; 1 mL/L water).

**Results:**

Supplementation with either probiotics (T3 and T4) significantly increased hen‐day egg production, egg weight and egg mass, while improving feed conversion ratio (FCR). The egg surface area and shell thickness were also significantly higher in the T3 and T4 groups. Among internal egg quality traits, albumen index, yolk weight and Haugh unit values were significantly improved by T3 and T4. Dietary T2 significantly reduced the white blood cell (WBC) and haemoglobin (Hb) concentrations, whereas T3 and T4 increased WBC counts and reduced blood cholesterol level (BCL) in laying hens. All treated groups exhibited significantly greater Newcastle disease virus (NDV) titre compared to T1. The T2 group showed the lowest total bacterial count, whereas T3 and T4 groups had significantly reduced coliform and enhanced lactic acid bacteria populations in the caecum.

**Conclusion:**

*Lactobacillus* alone or combined with *S. cerevisiae* can effectively replace antibiotics in commercial layer production by improving laying performance, egg quality, immunity and gut microbiota.

## Graphical abstract

Supplementation with *Lactobacillus plantarum* alone or combined with *Saccharomyces cerevisiae* improved laying performance, egg quality, immune response and caecal microbial balance by increasing beneficial lactic acid bacteria and reducing coliforms, demonstrating probiotics as a safe and sustainable alternative to antibiotic growth promoters in commercial layer production.

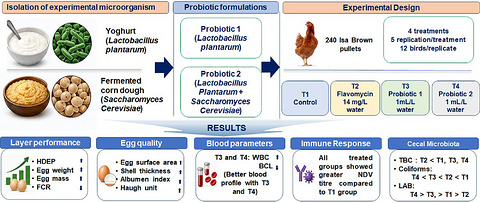

## 1 Introduction

Global demand for eggs is rising because they are an affordable source of high‐quality protein and essential nutrients. Per capita consumption has increased by 14% over the past decade globally (FAOSTAT 2025). However, in the push to increase production, intensive egg farming often overlooks bird welfare and health, leading to overcrowding, poor hygiene and inadequate nutrition that promote disease spread. To prevent infections and avoid economic losses, producers frequently rely on antibiotics, even without maintaining the proper dosing (Mateus et al. 2011; Saad and Ahmed 2018). The overuse of antibiotics and the presence of antibiotic residues in animal products are major contributors to the development of resistant microbial strains (Yin et al. 2023). Currently, the rate at which pathogens become tolerant to antibiotics is faster than the rate at which new antimicrobial drugs are produced. Therefore, many countries have banned the use of growth‐promoting antibiotics as feed additives to control the spread of antibiotic‐resistant strains. As withdrawal of antibiotics from feed has posed new hurdles, particularly in sustaining egg production and managing bacterial infections in chickens (Khalique et al. 2020), to maintain equilibrium between consumer's egg safety and animal health, developing safe and reliable antibiotic alternatives has become essential for the poultry sector. Numerous alternatives, such as probiotics, prebiotics, enzymes, organic acids and plant essential oils, have been extensively studied by researchers. Nonetheless, there is still a pressing need to identify the most effective and economically viable solutions as producers can face high costs with no assured return in terms of enhanced production performance.

In 2013, experts from the International Scientific Association for Probiotics and Prebiotics (ISAPP) defined probiotics as live microorganisms that, when consumed in adequate amounts, offer health benefits to the host (Ramos et al. 2020). The mode of action of probiotic includes generating beneficial metabolites such as short‐chain organic acids, stimulating the immune system, modifying gut microbial populations and competitively occupying receptor sites (Ahasan et al. 2015; Yin et al. 2023). The efficacy of probiotic supplementation is influenced by several factors, including host's physiological condition, the probiotic strain(s) used, the doses and the duration of administration (Gallazzi et al. 2008). Several previous studies have highlighted probiotics as a promising alternative to antibiotics, capable of improving laying performance (Obianwuna et al. 2022; Xu et al. 2022), egg quality (Lu et al. 2025; Ray et al. 2022) and immune response (Obianwuna et al. 2022; Yin et al. 2023), enhancing intestinal health and stabilizing gut flora (Obianwuna et al. 2022). *Lactobacilli*, a group of non‐pathogenic, Gram‐positive bacteria, are considered highly suitable probiotic candidates due to their prevalence as permanent members of the intestinal commensal microflora (Forte et al. 2016; Harimurti and Hadisaputro 2015). These organisms possess a wide spectrum of beneficial activities and are widely used in human and veterinary medicine (Dinev et al. 2018). Dietary supplementation with *Lactobacillus* spp. can improve the egg production, feed efficiency and eggshell quality (Gallazzi et al. 2008; Forte et al. 2016; Naseem et al. 2021). Additionally, *Lactobacillus* probiotics have been reported to stimulate immunity (Forte et al. 2016). *Saccharomyces cerevisiae* supplementation has also been observed to improve egg production, egg quality and feed conversion ratio (FCR) and enhance the absorption of nutrients and minerals in poultry (Brannan et al. 2024; Yalcin et al. 2008). Moreover, the synergistic effect of *Lactobacillus* and *Saccharomyces* has been proved (Istiqomah et al. 2019) and found positive effects feed digestibility (Wu et al. 2025), innate immunity and feed efficiency in poultry (Awais et al. 2019). However, to the best of our knowledge, no scientific studies have investigated the combined effects of *Lactobacillus plantarum* and *S. cerevisiae* in laying hens. Therefore, the objective of this study was to evaluate the effects of *Lactobacillus* alone and in combination with *S. cerevisiae* on production performance, egg quality, blood parameters, immune competence and intestinal microbial populations in layer chickens.

## 2 Materials and Methods

### 2.1 Preparation of Experimental Probiotic

In the present study, two experimental probiotic formulations were prepared: Probiotic 1 (*L. plantarum*) and Probiotic 2 (*L. plantarum* + *S. cerevisiae*). The isolation, identification and characterization of the microorganisms were carried out in Environmental Biotechnology Laboratory, Sher‐e‐Bangla Agricultural University (SAU), Dhaka, Bangladesh.

#### 2.1.1 Isolation and Identification of *Lactobacillus* spp

Fresh, locally produced yoghurt was used as the source of *Lactobacillus* spp. The sample was stored at 4°C and processed within 24 h of collection. *Lactobacillus* isolation procedure was carried out according to the method described by Somnath et al. (2017), with slight modifications. Briefly, 1 g of yoghurt was aseptically transferred into a sterile tube containing 9 mL of 0.85% (w/v) NaCl solution and homogenized using a stomacher for 1–2 min. Serial tenfold dilutions were prepared up to 10^−7^ using sterile saline solution. From each dilution, 0.1 mL aliquots were spread in duplicate onto de Man, Rogosa and Sharpe (MRS) agar plates, and incubated at 37°C for 24–48 h. After incubation, colonies exhibiting typical *Lactobacillus* morphology (pure white, round, smooth and entire edges) were observed and recorded. Colonies with distinct morphological characteristics were selected and streaked onto fresh MRS agar plates to obtain pure cultures. Sub‐culturing was repeated several times to ensure the purity of the isolates.

Identification of *L. plantarum* was done using morphological, cultural, physiological and biochemical characteristics (Kandler and Weiss 1986). The tests carried out were Gram staining, catalase and oxidase tests, glucose fermentation, NH_3_ from arginine, production of CO_2_ from Glucose, dextran from sucrose, acetate from glucose, motility test and the indole‐producing test.

#### 2.1.2 Isolation and Identification of *S. cerevisiae*

Fermented corn dough was obtained from a local food vendor and stored at 4°C until processing within 24 h. Approximately 10 g of dough was homogenized in 90 mL of a sterile diluent (0.1% peptone, 0.8% NaCl and pH 7.2). Serial tenfold dilutions were prepared up to 10^−6^. From each dilution, 0.1 mL aliquots were spread in duplicate onto malt agar supplemented with chloramphenicol (100 µg/mL) and tetracycline (50 µg/mL) to inhibit bacterial growth. The plates were incubated aerobically at 25°C for 7 days. Creamy‐white, raised and smooth colonies were selected and sub‐cultured on antibiotic‐free malt agar to obtain pure cultures. Sub‐culturing was repeated several times to ensure the purity of the isolates. Identification of *S. cerevisiae* was performed using morphological and biochemical characteristics, including Gram staining, carbohydrate fermentation (including maltose), nitrate reduction, and microscopic and macromorphological characterization.

#### 2.1.3 Molecular Characterization of *Lactobacilli* and *Saccharomyces*

Genomic DNA was extracted from purified isolates using boiling method following the method described by Heilig et al. (2002) and stored at −20°C for PCR analysis. For *L. plantarum*, PCR amplification was performed using universal bacterial primers 27‐F (forward 5′‐AGAGTTTGATCAATGGGTCAG‐3′) and 1492‐R (reverse 5′‐TACGGTTACCTTGTTACGATT‐3′) (Nübel et al. 1996). Identification of *S. cerevisiae* was carried out using ITS region primers ITS 1 (forward 5′‐CTTGGTCATTTAGAGGAAGT‐3′) and ITS 4 (reverse 5′‐TCCTGGGCTTATGATATGC‐3′) (Gardes and Bruns 1993). The PCR reactions were conducted in an Applied Biosystems 2720 thermal cycler (Roche, Mannheim, Germany) in a total reaction volume of 25 µL containing 1 µL template DNA, 10 pmol/µL of each primer, 12.5 µL of 2× PCR master mix and sterile distilled water up to 9.5 µL. For *Lactobacillus*, the PCR conditions consisted of an initial denaturation at 94°C for 3 min, followed by 32 cycles of denaturation at 94°C for 45 s, annealing at 55°C for 45 s, and extension at 72°C for 1 min, with final extension at 72°C for 6 min. For *S. cerevisiae*, the amplification protocol included an initial denaturation at 94°C for 5 min, followed by 32 cycles of 94°C for 45 s, annealing at 56°C for 45 s and extension at 72°C for 1 min and a final extension at 72°C for 10 min. The PCR products were separated by electrophoresis on a 1% agarose gel and visualized under UV illumination after ethidium bromide staining. The amplified DNA fragments were purified using an AccuPrep PCR Purification Kit (Cat. Nos. K‐3034 and K‐3034‐1, Bioneer Inc., Daejeon, Korea) according to the manufacturer's instruction. Purified amplicons were sequenced using an automated DNA sequencer with corresponding primers at Macrogen Inc., Seoul, Korea. The obtained sequences were compared with reference sequences in the GeneBank database using the basic local alignment search tool (BLAST) for species identification.

#### 2.1.4 Preparation of Inoculum

One gram of the isolated *L. plantarum* culture was inoculated into 10 mL of MRS broth in a sterile 15 mL screw‐cap tube and incubated anaerobically at 37°C for 48 h. Simultaneously, 1 g of *S. cerevisiae* was cultured into 10 mL of liquid yeast peptone dextrose (YPD) medium aerobically at 37°C for 48 h. Following incubation, both cultures were centrifuged at 5000 rpm for 5 min. The supernatants were carefully discarded, and the resulting cell pellets were washed twice with 1 mL phosphate buffered solution (PBS). The washed cell pellets were then resuspended in respective cryoprotectant (10% skim milk for *Lactobacillus* spp. and 10% (w/v) sterile skim milk supplemented with 5% (w/v) yeast extract for *S. cerevisiae*). The cultures were stored at −80°C for further use. Probiotic 1 was prepared using the *L. plantarum* (2 × 10^8^ colony forming unit [CFU]/mL), whereas Probiotic 2 was formulated by mixing *L. plantarum* and *S. cerevisiae* suspensions in a 1:1 (v/v) ratio, resulting in 1 × 10^8^ CFU/mL of *L. plantarum* and 1 × 10^7^ CFU/mL of *S. cerevisiae*.

### 2.2 Experimental Design

All animal handling procedures were approved by the Institutional Animal Care and Use Committee of Sher‐e‐Bangla Agricultural University (Dhaka, Bangladesh). A total of 240 16‐week‐old commercial Isa Brown pullets were procured from Kazi Farms Ltd., Bangladesh. The birds were randomly divided into four treatment groups comprising of 5 replications of 12 birds each. The treatments were T1 (control; no antibiotics or probiotics), T2 (antibiotic‐treated; flavomycin at 14 mg/L water), T3 (Probiotic 1 treated; 1 mL/L water) and T4 (Probiotic 2 treated; 1 mL/L water).

### 2.3 Bird Management

This study was conducted in an open‐sided poultry house using cage system. A 16 h/day lighting schedule was maintained using natural light and artificial light. The temperature in the room was maintained at 24°C to 28°C. Birds were acclimatized and fed a basal diet until 20 weeks of age. Data collection commenced at 24 weeks and continued until 50 weeks of age. The corn–soybean meal basal diet fed to laying hens was formulated to meet recommended nutrient requirements (NRC 1994). The composition and nutritional level of the basic diet and experimental diets are shown in Table 1.

**TABLE 1:** Ingredients and nutrient content of basal diet.

Ingredients (%)	Value	Nutrient profile	Value
Maize	41.00	Calculated composition	
Rice	19.50	ME (kcal/kg)	2878.2
Rice polishing	7.50	Crude protein (%)	17.93
Soybean meal	6.70	Analysed composition	
Cotton seed meal	1.50	Moisture (%)	10.53
Corn gluten 60%	6.10	Crude protein (%)	18.02
Fish meal	5.30	Ether extract (%)	3.13
Di‐calcium phosphate	2.70	Crude fibre (%)	4.67
Limestone	7.00	Total ash (%)	10.65
Molasses	2.20	Ca (%)	3.65
Vitamin‐mineral pre‐mix^a^	0.50	Available P (%)	0.44
Total	100.00		

*Note*: Values are expressed on an air‐dried basis.

^a^ Vitamin and mineral pre‐mix supplied per kilogram of diet: vitamin A, 14,000 UI; vitamin D3, 3000 UI; vitamin E, 30.00 mg, vitamin K, 2.00 mg; vitamin B1, 1.75 mg; vitamin B2, 12.00 mg; vitamin B6, 2.00 mg; vitamin B12, 0.015 mg; niacin, 35.00 mg; folic acid, 0.50 mg; pantothenic acid, 10.00 mg; Fe (FeCO3), 44.51 mg; Cu (CuSO4.5H2O), 58.95 mg; Mn (MnO), 193.5 mg; Zn (ZnO), 69.75 mg and Se (Na2SeO3) 6.00 mg.

### 2.4 Data Collection and Analyses

#### 2.4.1 Production Performance

The feed intake, egg production and egg weight were recorded daily throughout the experimental period (24–50 weeks). Then the average daily feed intake (ADFI), hen‐day egg production (%) egg mass and FCR were calculated.

#### 2.4.2 Egg Quality Parameters

At 44 weeks of age, 120 eggs per treatment (6 eggs/replicate) were randomly collected to measure the egg quality parameters. Egg weight, length and width were measured by using a digital scale and vernier caliper. Thereafter, egg shape index (Thompson et al. 1985) and surface area were calculated using the following formula:

Eggshapeindex=length/width

$$\text{Egg}\text{surface}\text{area}\;=\;4.67\;\times \;{\left(\text{egg}\text{weight}\right)}^{2/3}$$

After manual separation of egg components, the eggshell thickness was measured by eggshell thickness tester (ETG‐1601A, Robotmation, Tokyo, Japan). The weights of the shells were measured using a digital scale and eggshell index was calculated using to the followed equation (Ahmed et al. 2005):

Eggshellindex=(shellweight/eggsurfacearea)×100

The colour of the yolk was measured using a DSM Yolk colour fan. The height and width of the yolk and albumen were measured using a tripod micrometre. The albumen and yolk were also weighed to determine their relative weight. The albumen index (Doman et al. 2016), yolk index (Sharmin et al. 2021) and relative weights of yolk and albumen were calculated using the following equation:

$$\mathrm{Albumen}\;\mathrm{index}=\frac{\mathrm{Albumen}\;\mathrm{height}}{\left(\mathrm{Albumen}\;\mathrm{lenght}+\mathrm{Albumen}\;\mathrm{width}\right)/2}\;\times 100$$

Yolkindex=heightoftheyolk/widthofyolk×100

$${\mathrm{Components}}^{\prime }\;\mathrm{relative}\;\mathrm{weight}=\frac{\mathrm{Albumen}/\mathrm{yolk}\;\mathrm{weight}}{\mathrm{absolute}\;\mathrm{egg}\;\mathrm{weight}}\;\times \;100$$

Haugh unit was calculated from the records of albumen height and egg weight using the following formula proposed by Carter (1975):

$\text{HU}\;=\;100\;\log(H\;+\;7.56\;-\;1.7W^{0.37})$

where HU is the Haugh unit, *H* is the height of albumen (mL), and *W* is the egg weight in grams.

The proximate composition of eggs was measured according to AOAC (2002), and yolk cholesterol was extracted using the technique outlined by Washburn and Nix (1974), followed by quantification via the Libermann–Burchard colorimetric method.

#### 2.4.3 Humoral Immune Response

Immunization procedures consisted of a subcutaneous injection of Marek's disease (MD) vaccine at 1 day of age, followed by an eye‐intranasal administration of a combined vaccine (LaSota strain + H120 strain) at 7 days of age. At 14 days of age, birds received 0.5 mL of a trivalent vaccine (LaSota strain + M41 strain + H2 strain) via subcutaneous injection. On the 15 and 45 days post‐NDV vaccination, 3 birds/replications were randomly selected. Blood was collected from wing veins and serum was isolated and tested for the potency of antibodies for Newcastle disease. Antibody titres were determined using the haemagglutination inhibition (HI) assay. The HI titres were expressed as log_2_ of the reciprocal of the highest dilution exhibiting HI activity.

#### 2.4.4 Haematological Parameters

At the end of the experiment, three birds per replicate were randomly selected for blood sampling (5 mL from the wing vein). After collection, blood samples were transferred into sterile ethylenediaminetetraacetate (EDTA) universal bottles and thereafter taken to the laboratory at room temperature for haematological assay within 30 min of collection. Haematological variables were determined using the Auto Haematology Analyser (Model: BC‐2800 Vet). The red blood cell count (RBC), white blood cell count (WBC), haematocrit (HCT), haemoglobin (Hb), blood glucose level (BGL) and blood cholesterol level (BCL) were assessed according to the method described by Jain (1993).

#### 2.4.5 Determination of Intestinal Microbial Load

After blood collection, the selected birds were euthanized, and approximately 2 g of fresh intestinal content was collected from each bird and stored in a sterile ziploc bag. About 1 g of sample was diluted with 9 mL of sterilized physiological saline solution, and serial tenfold dilutions were prepared up to 10^−9^. A 1 mL aliquot from each dilution was spread out on plate count agar for total viable bacteria, McConkey agar for *Escherichia coli*, and MRS agar for *Lactobacillus* spp. and incubated for 24 h at 37°C. Following incubation, colonies were enumerated, and results were expressed as CFUs per gram of intestinal content.

### 2.5 Statistical Analysis

The experiment was carried out as a completely randomized design with four treatments. Data were subjected to ANOVA using the PROC IML ORPOL function of the Statistical Analysis System (SAS 2003). Pens were used as the experimental unit for production performance parameters and egg quality, whereas an individual bird served as the experimental unit for immunoglobulin concentrations and caecal bacterial population. Variability in the data was expressed as the SE, and a probability level of *p* ≤ 0.05 was considered statistically significant. Treatment means were computed with the LSMEANS option of the SAS program.

## 3 Results and Discussion

### 3.1 Production Performance

The effects of antibiotic and probiotic supplementation on the production performance of laying hens are presented in Table 2. Dietary treatments had no significant influence on feed intake or the age at 50% egg production (*p* > 0.05). However, hen‐day egg production was significantly higher in the T3 and T4 groups compared with the control and T2 group (*p* < 0.05). Egg weight and egg mass were also significantly higher in the T3 and T4 groups (*p* < 0.05). In addition, dietary supplementation of Probiotic 1 and Probiotic 2 significantly reduced FCR in the T3 and T4 groups relative to the control (*p* < 0.05). The effects of antibiotics on layer performance remain inconsistent. Some studies report improvements in egg production and FCR (Habib et al. 2017), whereas others found no significant benefits (Mahfuz et al. 2018). These discrepancies may depend on the antibiotic type, dosage and the birds’ health status. On the other hand, *Lactobacillus* spp. and *S. cerevisiae* have gained increasing attention as potential antibiotic substitutes for animal production due to their antibacterial properties and positive impacts on performance (Liu et al. 2025). The synergistic effects of *Lactobacillus* and *Saccharomyces* on feed digestibility and animal performance have also been demonstrated (Wu et al. 2025). Beyond this, studies examining their combined effects in layer chicken remain limited. In the present study, we found improvements in egg production, egg weight, egg mass and FCR in both the probiotic‐fed groups. Consistent with our results, Qiao et al. (2019) reported an enhanced laying rate in layers fed *L. plantarum* at concentration of 1.0 × 10^9^ CFU/mL. Farren et al. (2024) also reported higher egg production and egg weight in *L. plantarum* fed group compared to control. Alaqil et al. (2020) reported enhanced egg production, egg weight, egg mass and FCR in layer chickens supplemented with *L. acidophilus* at concentrations of 2 × 10^9^ and 3 × 10^9^ CFU/kg. Similarly, Panda et al. (2008) and Park et al. (2016) observed positive effects of lactic acid bacteria (LAB) on egg production in layer chickens, although they reported no significant effects on egg weight, which contrasts with our study. In contrary to our results, Onbaşılar et al. (2025) found no significant effects of *Lactobacillus‐*based probiotic on egg production or egg weight, although they did report a reduction in FCR in probiotic‐fed birds. Forte et al. (2016) and Naseem et al. (2021) also reported no effects of *Lactobacillus* probiotics on the production performance of laying hens. Brannan et al. (2024) observed higher feed intake and hen‐day egg production in the *S. cerevisiae* fed group without affecting the age at 50% production, partially supporting our findings. Variations among studies may be attributed to differences in probiotic strains, inclusion levels and the age of the hens. The improvement in production performance observed in the present study may be explained by modulation of the gut microbial balance through suppression of pathogen colonization and enhancing digestion and nutrient utilization (Yeo and Kim 1997; Aalaei et al. 2018; Lu et al. 2025).

**TABLE 2:** Comparative effects of antibiotic and two probiotics on production performance of laying hens.

Performance parameter	Dietary treatments				SEM	*p* value
	T1	T2	T3	T4		
Average daily feed intake (g)	111.13	111.52	112.13	113.08	0.35	0.230
Age at 50% production (day)	144.20	144.00	143.63	143.80	0.18	0.692
Hen‐day egg production (%)	90.27^b^	91.64^b^	93.04^a^	92.66^a^	0.34	0.045
Egg weight (g)	56.12^b^	56.06^b^	57.18^a^	57.19^a^	0.20	0.040
Egg mass (g)	50.87^b^	51.37^b^	53.20^a^	52.99^a^	0.30	0.002
FCR	2.18^a^	2.17^a^	2.11^b^	2.13^b^	0.01	0.045

*Note*: Means bearing different superscripts in a row differ significantly (*p *< 0.05); T1 = control group—no antibiotics or probiotics, T2 = antibiotic fed group, T3 = Probiotic 1 fed group (2 × 10^8^ CFU/mL *of Lactobacillus* spp.) and T4 = Probiotic 2 fed group (1 × 10^8^ CFU/mL of *Lactobacillus* spp. and 1 × 10^7^ CFU/mL of *S. cerevisiae*).

Abbreviations: FCR, feed conversion ratio; SEM, standard error of the mean.

### 3.2 Egg Quality Parameters

Table 3 summarizes the effects of antibiotic and probiotic supplementation on the external and internal egg quality traits. No significant differences were detected in shape index, shell proportion and shell index among the treatment groups (*p* > 0.05). However, egg surface area and shell thickness were significantly higher in the T3 and T4 groups compared to the T1 and T2 groups (*p* < 0.05). Regarding internal egg quality traits, albumen weight did not differ among the dietary treatments (*p* > 0.05). However, albumen index, yolk weight and Haugh unit values were significantly higher in T3 and T4 than in the T1 group (*p* < 0.05). In contrast, yolk index, yolk colour score and yolk cholesterol concentration were not influenced by dietary treatments (*p* > 0.05). Consistent with our findings, Habib et al. (2017) and Mahfuz et al. (2018) also reported no significant effects of dietary flavomycin on egg quality parameters. The improved shell thickness observed in the probiotic supplemented groups align with the findings of Xu et al. (2022). This improvement in eggshell quality may be attributed to the ability of probiotics to enhance calcium assimilation and retention in the serum of laying birds, thereby facilitating calcium deposition in the shell gland (Obianwuna et al. 2022). Additionally, we observed increased albumen index, yolk weight and Haugh units, which is also supported by previous research using *Lactobacillus*‐based probiotics as feed additives (Farren et al. 2024; Gallazzi et al. 2008; Xu et al. 2022). We did not find any research specifically examining the combined effects of *Lactobacillus* and *S. cerevisiae* in layer chickens to make direct comparisons. The improvement in albumen quality due to dietary probiotics may be attributed to enhanced nutrient digestibility and subsequent increases in protein synthesis (Lei et al. 2013). Consistent with our result, Xiang et al. (2019) also found no effects of probiotics on yolk cholesterol content. In contrast, Forte et al. (2016) found no significant effects of *Lactobacillus*‐based probiotic on the egg quality parameters in organic laying hens. The variation among studies may be due to differences in bird type, probiotic species, strain characteristics and dosage levels.

**TABLE 3:** Comparative effects of antibiotic and two probiotics on egg quality of laying hens.

Performance parameter	Dietary treatments				SEM	*p* value
	T1	T2	T3	T4		
External egg quality traits						
Shape index (%)	72.06	72.29	72.58	73.78	0.29	0.158
Egg surface area (cm^2^)	68.45^b^	69.33^b^	70.25^a^	70.26^a^	0.21	0.001
Shell proportion (%)	10.18	10.16	10.16	10.21	0.21	0.677
Shell thickness (mm)	0.34^b^	0.34^b^	0.37^a^	0.37^a^	0.003	0.007
Eggshell index (g/100 cm^2^)	14.82	14.87	14.47	14.54	0.09	0.303
Internal egg quality						
Albumen weight (g)	37.27	37.23	37.57	37.43	0.09	0.593
Albumen index	8.91^b^	9.61^ab^	10.08^a^	10.14^a^	0.11	0.046
Yolk weight (g)	14.18^b^	14.34^b^	15.02^a^	15.04^a^	0.11	0.001
Yolk index	42.39	42.13	42.71	43.36	0.35	0.658
Yolk colour score	7.42	7.64	7.52	7.48	0.05	0.396
Yolk cholesterol (mg/g)	11.27	11.47	11.23	11.38	0.06	0.458
Haugh unit	83.80^b^	84.16^ab^	84.28^a^	84.31^a^	0.07	0.003

*Note*: Means bearing different superscripts in a row differ significantly (*p *< 0.05); T1 = control group—no antibiotics or probiotics, T2 = antibiotic fed group, T3 = Probiotic 1 fed group (2 × 10^8^ CFU/mL *of Lactobacillus* spp.) and T4 = Probiotic 2 fed group (1 × 10^8^ CFU/mL of *Lactobacillus* spp. and 1 × 10^7^ CFU/mL of *S. cerevisiae*).

Abbreviation: 3SEM, standard error of the mean.

### 3.3 Haematological Parameters

Blood parameters are commonly used to assess the level of stress in animals. Table 4 presents the haematological profile of laying hens in response to dietary antibiotic and probiotic supplementation. Dietary treatments did not significantly affect RBC count, HCT or BGL (*p* > 0.05). However, dietary antibiotic supplementation significantly reduced WBC and Hb concentrations of laying hens (*p* < 0.05). In contrast, both probiotic supplementations significantly increased WBC counts with significantly reduced BCL in laying hens (*p* < 0.05). Consistent with our study, Bhuiyan et al. (2021) and AL‐Mayah and AL‐Ahmed (2005) also reported reduced WBC and Hb concentrations in chickens fed antibiotics. Miah et al. (2025) reported higher WBC concentrations in indigenous naked neck chickens fed diets containing *Clostridium butyricum* and *L. plantarum*, which aligns with our results. Maoba et al. (2021) likewise observed significantly higher Hb levels in Boschveld chickens supplemented with *S. cerevisiae* probiotic. According to Ashayerizadeh et al. (2011), dietary supplementation of probiotic can lower blood cholesterol concentrations compared with birds fed antibiotic, prebiotic or basal diets. Alaqil et al. (2020) also reported reduced plasma cholesterol in laying hens fed *L. acidophilus*, which supports our finding. The hypocholesterolemic activity of *Lactobacillus* and *Saccharomyces* may be attributed to their ability to assimilate cholesterol into the cellular membranes and utilize it for metabolism, thereby reducing intestinal cholesterol absorption and inhibiting cholesterol synthesis (Ziarno 2008; Saikia et al. 2017).

**TABLE 4:** Comparative effects of antibiotic and two probiotics on blood profiles of laying hens.

Performance parameter	Dietary treatments				SEM	*p* value
	T1	T2	T3	T4		
RBC (×10^6^/mm^3^)	3.26	3.25	3.24	3.26	0.03	0.998
WBC (×10^3^/mm^3^)	21.52^b^	20.22^c^	22.29^a^	22.56^a^	0.56	<0.0001
HCT (%)	31.24	32.33	32.46	32.12	0.21	0.138
Hb (g/dL)	6.38^a^	6.12^b^	6.42^a^	6.45^a^	0.05	0.005
BGL (mmol/L)	12.62	12.11	12.25	12.48	0.39	0.166
BCL (mg/dL)	142.56^a^	140.12^a^	130.11^b^	116.33^c^	2.53	<0.0001

*Note*: Means bearing different superscripts in a row differ significantly (*p *< 0.05); T1 = control group—no antibiotics or probiotics, T2 = antibiotic fed group, T3 = Probiotic 1 fed group (2 × 10^8^ CFU/mL *of Lactobacillus* spp.) and T4 = Probiotic 2 fed group (1 × 10^8^ CFU/mL of *Lactobacillus* spp. and 1 × 10^7^ CFU/mL of *S. cerevisiae*).

Abbreviations: BCL, blood cholesterol level; BGL, blood glucose level; HB, haemoglobin; HCT, haematocrit; RBC, red blood cell; SEM, standard error of the mean; WBC, blood cell count.

### 3.4 Immunological Response

**FIGURE 1:**
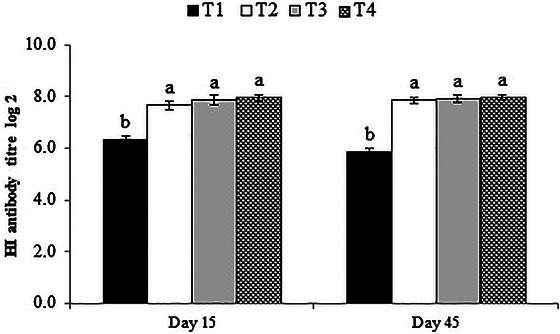
Comparative effects of antibiotic and two probiotics on HI antibody titres against Newcastle disease virus (NDV). a,bMeans bearing different superscripts in a day period differ significantly (p < 0.05); T1 = control group—no antibiotics or probiotics, T2 = antibiotic fed group, T3 = Probiotic 1 fed group (2 × 108 CFU/mL of Lactobacillus spp.) and T4 = Probiotic 2 fed group (1 × 108 CFU/mL of Lactobacillus spp. and 1 × 107 CFU/mL of S. cerevisiae). HI, haemagglutination inhibition.

Figure 1 displays the HI antibody titres against Newcastle disease virus (NDV) in the birds receiving the experimental diets. All treated groups exhibited significantly greater levels of NDV titre (*p *< 0.05) compared with the control group. Consistent with our study, Daneshmand et al. (2012) and Huang et al. (2006) also reported that antibiotic inclusion enhanced the antibody response to NDV as compared to control. Similarly, Forte et al. (2016) and Gharajalar et al. (2020) observed higher HI antibody titre in chickens supplemented with *L. acidophilus*. Yin et al. (2023) further demonstrated that *L. plantarum* GX17 improved the antibody potency of yellow‐feathered broilers immunized with Newcastle disease vaccine and avian influenza vaccine. Furthermore, Soren et al. (2024) reported higher HI antibody titres in broilers fed *S. cerevisiae* fermentation product at 28 days of age. During stress, activation of the hypothalamic pituitary–adrenal axis leads to elevated corticosterone secretion, which can suppress humoral immunity and reduce antibody production (Honda et al. 2015). Probiotics, such as *Lactobacillus* and *Saccharomyces*, have been shown to reduce corticosterone levels (Meimandipour et al. 2010; Feng et al. 2025), which may contribute to the increased antibody titre against NDV observed in the present study. The combination of these two probiotics may exert a synergistic effect by simultaneously reducing stress hormone levels and improving the immune response to viral challenges like NDV.

### 3.5 Intestinal Microbial Status

Table 5 presents the effects of dietary treatments on intestinal microbial load of laying hens supplemented with antibiotics and probiotics. The antibiotic‐treated group (T2) showed a significantly lower total bacterial count compared to the other groups (*p* < 0.05). Interestingly, both probiotic‐treated groups (T3 and T4) significantly reduced the concentration of coliform bacteria (*p* < 0.05) and increased the population of LAB in the intestine compared to T1 and T2 (*p* < 0.05). Our findings are supported by Lu et al. (2025), who reported improvement in caecal microbiota in laying hens supplemented with a complex probiotics (*Bacillus subtilis* ≥1 × 10^9^ CFU/g; LAB ≥2 × 10^7^ CFU/g and *Saccharomyces* ≥1 × 10^7^ CFU/g). Similarly, Park et al. (2016) reported reduced coliform counts in the excreta of laying hens fed diet supplemented with *Enterococcus faecium*. Hassanein and Soliman (2010) also reported increased ileal *Lactobacilli* counts and decreased coliform counts in laying hens fed 0.4%–1.6% *S. cerevisiae*. Soren et al. (2024) found reduced *E. coli* concentrations in the pre‐caecal content of broilers supplemented with *S. cerevisiae*. Chickens host diverse microbial communities, particularly in the gastrointestinal tract, which play a critical role in host health and productive performance (Beldowska et al. 2023). Probiotics, being active beneficial microorganisms, can modulate intestinal microbiota composition by enhancing the proliferation of beneficial bacteria and suppressing pathogenic species, thereby improving overall poultry health (Ahmad et al. 2022; Krysiak et al. 2021). The higher concentration of LAB and lower coliforms counts in the probiotic treated groups suggest a favourable modulation of the gut microbiota, which may contributed to the enhanced HI antibody titres against NDV (Qiao et al. 2021).

**TABLE 5:** Comparative effects of antibiotic and two probiotics on caecal microbial population in layers (log _10_ CFU/g).

Performance parameter	Dietary treatments				SEM	*p* value
	T1	T2	T3	T4		
Total bacterial count	6.48^a^	6.26^b^	6.50^a^	6.62^a^	0.04	0.011
Coliforms	3.59^a^	3.48^ab^	3.40^b^	3.39^b^	0.03	0.033
Lactic acid bacteria	5.44^c^	5.01^b^	6.13^a^	6.16^a^	0.12	<0.0001

*Note*: Means bearing different superscripts in a row differ significantly (*p *< 0.05); T1 = control group—no antibiotics or probiotics, T2 = antibiotic fed group, T3 = Probiotic 1 fed group (2 × 10^8^ CFU/mL *of Lactobacillus* spp.) and T4 = Probiotic 2 fed group (1 × 10^8^ CFU/mL of *Lactobacillus* spp. and 1 × 10^7^ CFU/mL of *S. cerevisiae*).

Abbreviation: SEM, standard error of the mean.

## 4 Conclusion

The study demonstrated that dietary supplementation with *Lactobacillus*‐based probiotic alone in combination with *Saccharomyces* effectively improved laying performance and egg quality in laying hens without affecting feed intake. Additionally, both probiotics enhanced the immune function by increasing WBC concentrations, elevating HI antibody titres (log_2_) against Newcastle disease, and promoting a healthier gut microbial environment. Overall, both probiotic performed better than antibiotics across most evaluated parameters. Therefore, the probiotics used in this study may serve as effective and sustainable alternatives to conventional antibiotic growth promoters in layer chicken diets.

## Author Contributions

**Md. Jahangir Alam**: supervision, conceptualization, methodology, data curation, project administration, funding acquisition, review and editing, resources. **Sonia Tabasum Ahmed**: methodology, data curation, project administration, validation; formal analysis, writing – original draft. **Md. Saiful Islam**: conceptualization, investigation, methodology, data curation, formal analysis. **Mahfuzul Islam**: investigation, methodology, review and editing, data curation. **Md. Mahabbat Ali**: investigation, methodology, editing. **Sang‐Suk Lee** : methodology, formal analysis.

## Ethics Statement

All experimental protocols complied with the guidelines approved by the Animal Ethics Committee of Sher‐e‐Bangla Agricultural University (Dhaka, Bangladesh). This study involved only non‐invasive procedures on laying hens. The hens were maintained under standard husbandry practices during the trial period.

## Conflicts of Interest

The authors declare no conflicts of interest.

## Data Availability

Readers can request the data supporting the findings of this study by contacting the email address listed.
